# Accurate staging of non-metastatic colon cancer with CT: the importance of training and practice for experienced radiologists and analysis of incorrectly staged cases

**DOI:** 10.1007/s00261-022-03573-7

**Published:** 2022-07-07

**Authors:** S. van de Weerd, E. Hong, I. van den Berg, J. W. Wijlemans, J. van Vooren, M. W. Prins, F. J. Wessels, B. C. Heeres, S. Roberti, J. Nederend, J. H. J. M. van Krieken, J. M. L. Roodhart, R. G. H. Beets-Tan, J. P. Medema

**Affiliations:** 1 Laboratory for Experimental Oncology and Radiobiology, Center for Experimental and Molecular Medicine, Cancer Center Amsterdam, Amsterdam UMC, University of Amsterdam, Amsterdam, The Netherlands; 2 Department of Pathology, Radboud University Medical Centre, Nijmegen, The Netherlands; 3 Oncode Institute, Amsterdam UMC, University of Amsterdam, Amsterdam, The Netherlands; 4 Department of Radiology, The Netherlands Cancer Institute, Amsterdam, The Netherlands; 5 Department of Radiology, Seoul National University Hospital, Seoul, South Korea; 6 GROW School for Oncology and Developmental Biology, Maastricht University Medical Center, Maastricht, The Netherlands; 7 Department of Surgery, Erasmus MC, University Medical Center Rotterdam, Rotterdam, The Netherlands; 8 Department of Medical Oncology, University Medical Center Utrecht, Utrecht University, Utrecht, The Netherlands; 9 Department of Radiology, University Medical Center Utrecht, Utrecht University, Utrecht, The Netherlands; 10 Department of Epidemiology and Biostatistics, The Netherlands Cancer Institute, Amsterdam, The Netherlands; 11 Department of Radiology, Catharina Hospital, Eindhoven, The Netherlands

**Keywords:** Colon cancer, Radiology, Learning curve, Computed tomography, Neoadjuvant therapy

## Abstract

**Purpose:**

To investigate whether locoregional staging of colon cancer by experienced radiologists can be improved by training and feedback to minimize the risk of over-staging into the context of patient selection for neoadjuvant therapy and to identify potential pitfalls of CT staging by characterizing pathologic traits of tumors that remain challenging for radiologists.

**Methods:**

Forty-five cases of stage I-III colon cancer were included in this retrospective study. Five experienced radiologists evaluated the CTs; 5 baseline scans followed by 4 sequential batches of 10 scans. All radiologists were trained after baseline scoring and 2 radiologists received feedback. The learning curve, diagnostic performance, reader confidence, and reading time were evaluated with pathologic staging as reference. Pathology reports and H&E slides of challenging cases were reviewed to identify potential pitfalls.

**Results:**

Diagnostic performance in distinguishing T1-2 vs. T3-4 improved significantly after training and with increasing number of reviewed cases. Inaccurate staging was more frequently related to under-staging rather than over-staging. Risk of over-staging was minimized to 7% in batch 3–4. N-staging remained unreliable with an overall accuracy of 61%. Pathologic review identified two tumor characteristics causing under-staging for T-stage in 5/7 cases: (1) very limited invasive part beyond the muscularis propria and (2) mucinous composition of the invading part.

**Conclusion:**

The high accuracy and specificity of T-staging reached in our study indicate that sufficient training and practice of experienced radiologists can ensure high validity for CT staging in colon cancer to safely use neoadjuvant therapy without significant risk of over-treatment, while N-staging remained unreliable.

## Introduction

Interest in neoadjuvant therapy for locally advanced colon cancer patients has been increasing rapidly during the recent years. Possible advantages are early eradication of micrometastases, the possibility of response monitoring, and an increased complete resection rate. Recent studies on neoadjuvant therapy for colon cancer patients, like the FOxTROT (chemotherapy) and NICHE trial (immunotherapy), have highlighted its great potential [1, 2]. The recently started CONNECTION-II trial investigates the predictive value of the consensus molecular subtypes in terms of response to neoadjuvant chemotherapy in radiological high-risk colon cancer patients, defined as T3-4 tumors [3].

One important concern of neoadjuvant therapy is to avoid unnecessary treatment of low-risk patients by adequately selecting high-risk colon cancer patients using accurate radiologic staging. The role of the pre-treatment CT in colon cancer has therefore extended from detecting distant metastases to also include locoregional staging. A meta-analysis on the accuracy of radiological staging showed a sensitivity and specificity of 96 and 70%, respectively, for distinguishing T1-2 from T3-4 [4]. The assessment of nodal involvement was much less reliable with a pooled sensitivity and specificity of 78% and 68%, respectively. The poor performance in N-staging has led to the fact that radiologic patient selection for neoadjuvant therapy is often solely based on T-staging [2, 3, 5–7].

The anatomical orientation of the colon and the constant movement of the bowel due to peristalsis make adequate locoregional CT staging of colon cancer especially challenging. Accurate CT staging might involve a steep learning curve and may require considerable experience and practice. We recently showed the presence of a learning curve for locoregional staging in senior trainees, with a significant increase in their performance to distinguish T1-2 from T3-4 and an inflection point at 38 cases, while no improvement was seen for N-staging [8]. However, in clinical practice, selection of high-risk patients for neoadjuvant treatment of colon cancer will likely be performed by experienced radiologists, and learning curves in experienced radiologists have not been evaluated. Moreover, not much is known about the specific radiologic or pathologic characteristics of colon cancer cases in which accurate distinction between high and low risk is difficult. Another important aspect in patient selection for neoadjuvant therapy is the reported confidence of the radiologist in distinguishing high-risk colon cancer, since uncertainty about radiologic staging might cause reluctance in patient selection for neoadjuvant therapy.

Considering the increasing importance of locoregional staging of colon cancer, we investigated whether radiologic CT staging and confidence of experienced radiologists can be improved by repetition and by receiving training and feedback. We were particularly interested in the risk of over-staging and whether this risk could be reduced in order to minimize unnecessary treatment with neoadjuvant therapy in low-risk patients. We further aimed to characterize the cases where radiologic interpretation remains challenging. These results can be used for optimal training of radiologists in clinical trials and in daily practice.

## Material and methods

### Study population

We included patients who underwent presurgical CT followed by surgical resection of colon cancer within the MATCH database. The MATCH study is a prospective multicenter cohort study including patients with stage I-III colorectal cancer from 2007 until December 2017 in seven hospitals in the region of Rotterdam, the Netherlands [9]. The MATCH study was approved by the Erasmus MC medical ethics review board (MEC-2007-088) and all patients provided written informed consent.

Inclusion criteria were colon cancer patients who underwent pre-treatment CT with slice thickness of 3 mm for adequate evaluation. Exclusion criteria were patients with rectal cancers, small sized colon tumor lesions which could not be visualized on CT, poor image quality, and patients who received neoadjuvant therapy. These selection criteria left us with scans from one of the seven centers from the MATCH study, with slice thickness as the main discriminating criterion. From this center, 45 cases were consecutively selected, in sequential order of the original trial. Next, patients were divided evenly into 5 batches (1 baseline batch of 5 cases and 4 batches of 10 cases), so that each batch contained a similar variety of pathologic TNM stages. The baseline batch containing 5 cases was used to assess the baseline accuracy of radiologic staging, the other batches were utilized for the evaluation of the learning curves.

#### CT scans of the included colon cancer patients

All patients were kept on nil per os for 2–4 h, and bowel preparation was not performed before the CT scan. CT scans were performed with a 16-channel CT scanner (Aquilion, Canon, Tokyo, Japan). All patients underwent preoperative abdominal CT with iodine-based intravenous contrast (3–5 ml/s, total amount of 90–150 ml, followed by bolus injection of 30 cc normal saline) in portal-venous phase at 70 s delay. Images from all CT scanners were reconstructed at 3-mm slice thickness.

#### Image interpretation

A total of 5 board-certified radiologists (all with 5 + years of experience in abdominal images, of which two with 10 + years) from two separate academic hospitals participated in this study. Readers were blinded to all clinical and pathological data, except for the tumor location. The following imaging features were recorded independently: (1) T-staging of tumor and reader’s confidence using a 0 to 4 scale with 4 as the most confident and 0 as the least confident; (2) N-staging and reader’s confidence; and (3) reading time in seconds. T1-2 tumors were defined as an intraluminal mass with no evidence of extraluminal extension or bowel wall deformation. T3 tumors were defined as tumors with a smooth or nodular, not spiculated, extension beyond the normal delineation of the bowel wall. T4 tumors were defined as tumors extending into adjacent peritoneum or growing into other adjacent tissues or organs. A lymph node with metastasis was defined as a lymph node with a short axis diameter over 8 mm [10].

First, all readers scored 5 scans without any instructions or training to assess the baseline accuracy, followed by a 45-min lecture on colon cancer CT staging provided by an experienced board-certified radiologist (EKH, with over 8 years of experience, who evaluated over 600 cases of colon cancer staging), under the supervision of a senior faculty member (RBT, with over 20 years of experience in abdominal CT imaging). This lecture covered the principles and criteria of colon cancer staging, including radiologic and pathologic definition of T- and N-staging of colon cancer. Next, readers were provided with 1 batch of 10 scans per week, with a total of 4 batches. All scans were additionally scored by EKH to assess the expert radiologist performance.

The readers were randomly divided into the feedback group (*n* = 2) or the no-feedback group (*n* = 3). Each group contained one reader with more than 10 years of experience. The readers in the feedback group were provided with histopathological data after interpretation of each batch, allowing a comparison with their radiological findings.

#### Pathologic interpretation

Routine pathologic staging was used as the reference standard. Processing of the specimen was performed according to local institutional protocols. The national pathology database (i.e., nationwide network and registry of histo- and cytopathology in the Netherlands, PALGA) protocol was used for standardized reporting of the histopathological information [11]. Pathologic T- and N-staging were utilized for analysis. Additionally, we performed a thorough review of the pathology reports in cases scored incorrectly by 3 or more radiologists and/or the expert (regarding T-stage) or scored incorrectly by 4 or more radiologists and/or the expert (regarding N-stage). Hematoxylin and eosin (H&E)-stained sections from the challenging T-stage cases were re-evaluated by an experienced pathologist (HK).

### Statistics

Accuracies, sensitivities, specificities, and positive and negative predictive values (PPV and NPV) of the readers in differentiating T1-2 from T3-4 and N0 from N1-2 colon cancer were evaluated both by batch and overall. To assess improvement in these quantities with increased reader experience, the difference in performance between groups of batches was compared between batch 0 and batches 1–4, between batches 0–1 and 2–4, between batches 0–2 and 3–4, and finally between batches 0–3 and batch 4. Testing for significance of the difference between groups of batches was done using Wald tests with robust standard errors obtained from logistic generalized estimating equations (GEE) models with the group of batches as the only independent variable, an independence working correlation structure, and patient id as the clustering variable. These analyses were repeated with only the post-training batches, i.e., batches 1–4.

Averages for confidence and reading time were obtained both separately by batch, and overall. Since additional radiologic features were scored in batches 1–4, the reading time for batch 0 was not comparable and was therefore not studied. Because individual batches were not large enough to fit ordinal GEE models, we treated confidence as a continuous score and tested for differences in mean confidence between feedback groups using Wald tests with robust standard errors obtained from standard linear GEE models. For these models, feedback group was the independent variable, we used an exchangeable working correlation structure, and patient id was used for clustering. Testing for differences of mean reading time between feedback groups was done in the same way, with reading time as the dependent variable instead of confidence. For confidence, groups of batches were also compared to assess the effect of increased reader experience, and an overall difference in confidence between correctly and erroneously staged cases was assessed using a GEE model with correctness of staging (yes/no) as the independent variable. Coherence between pathologic and radiologic staging was assessed using Cohen’s kappa.

Finally, learning curves were obtained for T-staging and N-staging using logistic GEE models with individual reader intercepts and a separate learning effect for both feedback groups, and an exchangeable working correlation structure.

All statistical analyses were performed using IBM SPSS Statistics software (version 28), R version 4.1.1 and MedCalc version 19.1.3. *P* values < 0.05 were considered statistically significant.

## Results

Forty-five cases were selected for this study. After reviewing the pathology reports, one case in batch 2 was removed because it was a rectal tumor that received neoadjuvant radiotherapy. The final cohort consisted of 44 colon cancer patients with a median age of 70.5 (interquartile range 63.0–77.0 years) (Table 1). Of the 44 tumors, 17 (38.6%) were located in the cecum, 4 (9.1%) in the ascending colon, 3 (6.8%) in the transverse colon, 1 (2.3%) in the distal transverse colon, 3 (6.8%) in the descending colon, and 16 (36.4%) in the sigmoid colon.

**Table 1:** Patient demographics and tumor characteristics

Characteristic	Number (%)
Age (median) IQR	70.5 63.0–77.0
*Gender*	
Male Female	21 (47.8) 23 (52.2)
*Tumor Location*	
Cecum Ascending colon Transverse colon Distal transverse colon Descending colon Sigmoid colon	17 (38.6) 4 (9.1) 3 (6.8) 1 (2.3) 3 (6.8) 16 (36.4)
*Pathologic T-stage*	
pT1 pT2 pT3 pT4	3 (6.8) 12 (27.3) 24 (54.5) 5 (11.4)
*Pathologic N-stage*	
pN0 pN1 pN2	28 (63.6) 11 (25.0) 5 (11.4)

*IQR * Interquartile range

## T-staging

The diagnostic performance of all readers in distinguishing T1-2 vs. T3-4 colon cancer is depicted in Table 2. Accuracy of the 5 readers in differentiating T1-2 vs. T3-4 colon cancer improved from 62% (74/120) in batches 0–2 to 81% (81/100) in batches 3–4 (*p* = 0.027). When only considering post-training batches, accuracy improved significantly between batches 1–2 and 3–4 (*p* = 0.042). These results indicate that staging accuracy improved after training and continued to improve after multiple batches. No further significant improvement was observed.

**Table 2:** Diagnostic performance of all radiologists in distinguishing T1-2 vs. T3-4 and N0 vs. N1-2 with increasing number of evaluated cases

Batch	Accuracy	Sensitivity	Specificity	PPV	NPV
*T-staging (T1-2 vs. T3-4)*					
0	60 (15/25)	87 (13/15)	20 (2/10)	62 (13/21)	50 (2/4)
1	58 (29/50)	57 (20/35)	60 (9/15)	77 (20/26)	38 (9/24)
2	67 (30/45)	63 (19/30)	73 (11/15)	83 (19/23)	50 (11/22)
3	82 (41/50)	86 (30/35)	73 (11/15)	88 (30/34)	69 (11/16)
4	80 (40/50)	77 (23/30)	85 (17/20)	88 (23/26)	71 (17/24)
All	70 (155/220)	72 (105/145)	67 (50/75)	81 (105/130)	56 (50/90)
*N-staging (N0 vs. N1-2)*					
0	48 (12/25)	20 (1/5)	55 (11/20)	10 (1/10)	73 (11/15)
1	66 (33/50)	52 (13/25)	80 (20/25)	72 (13/18)	63 (20/32)
2	51 (23/45)	53 (8/15)	50 (15/30)	35 (8/23)	68 (15/22)
3	72 (36/50)	65 (13/20)	77 (23/30)	65 (13/20)	77 (23/30)
4	62 (31/50)	60 (9/15)	63 (22/35)	41 (9/22)	76 (22/28)
All	61 (135/220)	55 (44/80)	65 (91/140)	47 (44/93)	72 (91/127)

*Note* numbers are in percentages, absolute numbers are given between parentheses.

*PPV* Positive predictive value*, NPV N*egative predictive value

Specificity reached a level of 80% (28/35) in batch 3–4, compared to 55% (22/40) in batches 0–2. The improvement in specificity was significant for batch 0 vs. 1–4 (*p* < 0.001). Sensitivity appeared to improve (e.g., from 65% (52/80) in batches 0–2 to 82% (53/65) in batches 3–4), but these results were not statistically significant.

A trend was seen in the improvement of the positive predictive value (PPV) with an increased number of reviewed cases. PPV reached 88% (53/60) in batches 3–4, as opposed to 74% (52/70) in batch 0–2 (*p* = 0.242). No significant differences were seen in negative predictive value when comparing different combinations of batches.

**Fig. 1:**
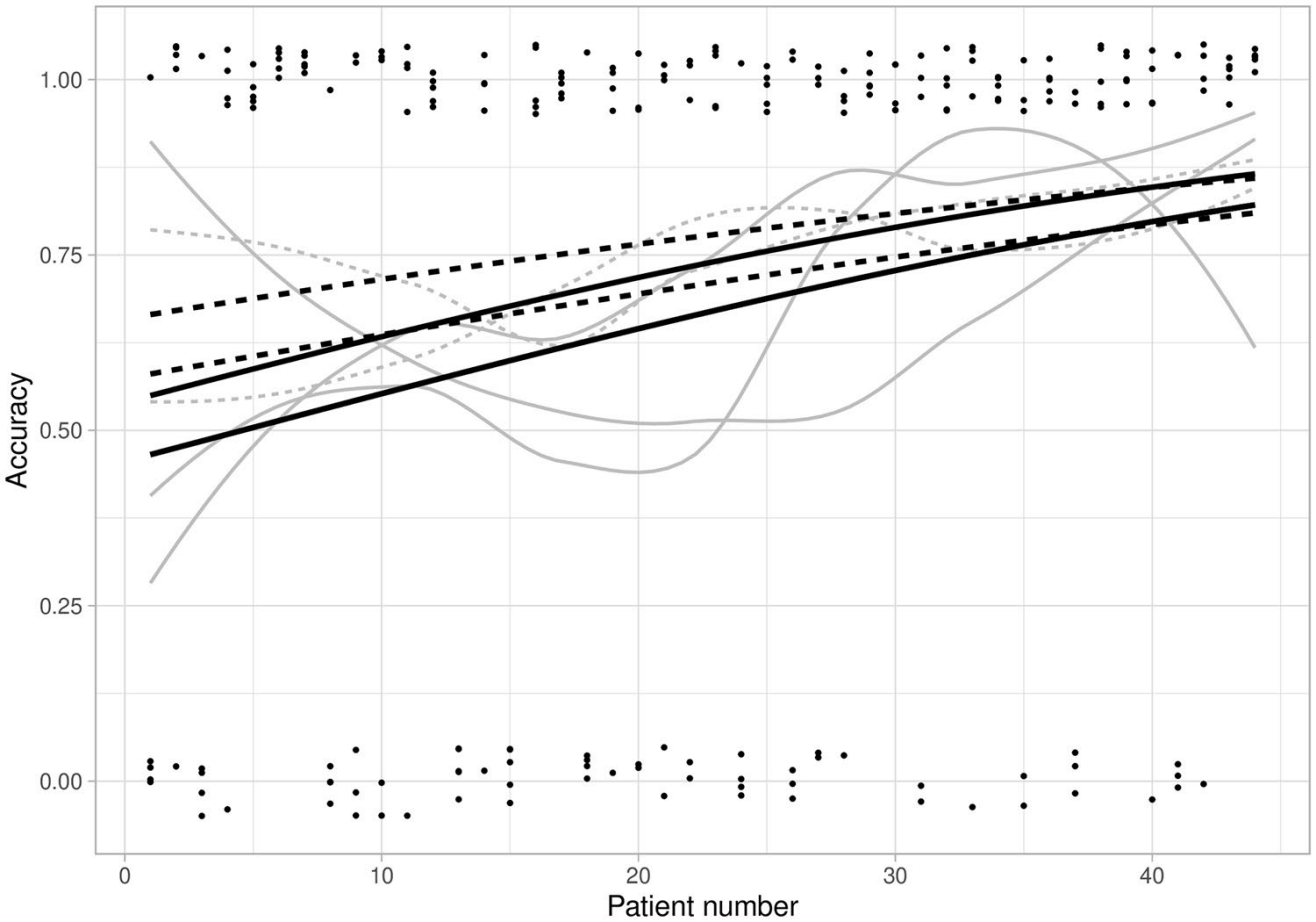
Learning curves for T-staging (T1-2 vs. T3-4) of colon cancer, separately for each radiologist. Accuracy of radiologists who received feedback (dashed line) and did not receive feedback (solid line) is presented. Accuracy was plotted and fitted using generalized estimating equations logistic regression models (black lines, the lower black line represents the same learning curve for two radiologists). Dots represent the number of correct (around 1.00) or incorrect (around 0.00) staged readings, and gray lines are non-parametric smoothed curves

**Fig. 2:**
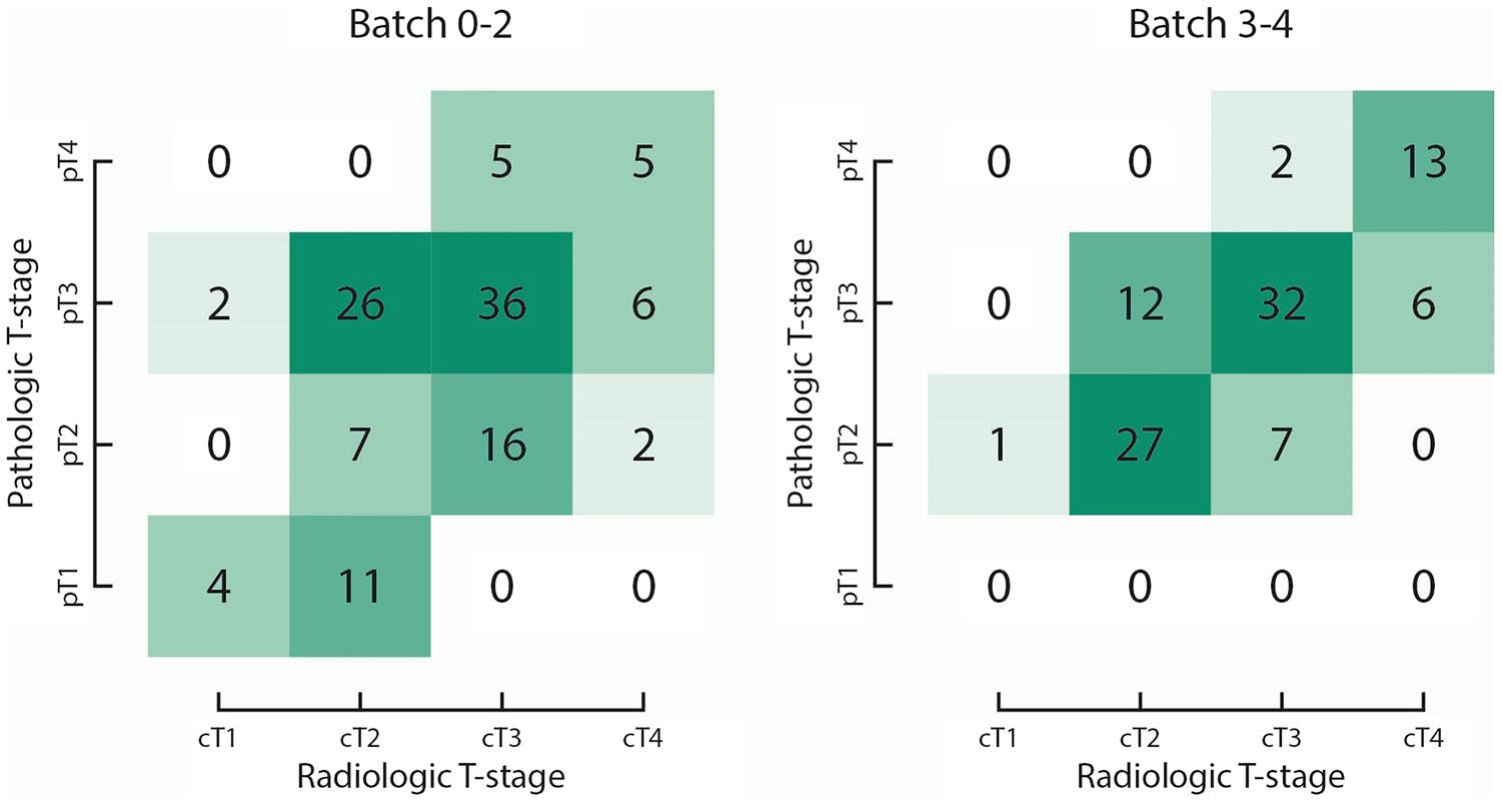
Contingency tables with radiologic T-stage and pathologic T-stage depicted for batch 0–2 and batch 3–4. All cases were scored by 5 radiologists, adding up to 120 readings within batch 0–2 and 100 readings in batch 3–4. The numbers represent readings. For example, 14 pT3 patients were scored in batch 0–2 by 5 radiologists, adding up to 70 readings of which 36 were correctly scored as cT3

Although we aimed to form batches with a similar variety of pathologic TNM stages, a discrepancy was present in the number of pT4 tumors between batches 0–2 and 3–4 (i.e., 10 cases vs. 15 cases). Since pT4 tumors might be easier to recognize as high-risk tumors on CT and were indeed all properly classified as high risk, we repeated the analyses after exclusion of all pT4 tumors. With this approach, accuracy still improved from 58% (64/110) in batches 0–2 to 78% in batches 3–4 (66/85). The observed improvement remained statistically significant (*p* = 0.035) (results not shown). The learning curve for accuracy in distinguishing T1-2 vs. T3-4 colon cancer is depicted in Fig. 1. Staging accuracy was initially higher, but improved more gradually for readers who received feedback. This difference was, however, not significant (results not shown). The concordance between radiologic T-stage and pathologic T-stage improved between batch 0–2 (*κ* = 0.26) and batch 3–4 (*κ* = 0.63) (Fig. 2).

We were particularly interested in the risk of over-staging and whether this risk could be minimized with training and repetition. Overall, incorrect staging was more frequently related to under-staging rather than over-staging. Importantly, the risk of over-staging decreased with increasing number of reviewed cases. In batches 0–2, 22 out of 40 (55%) low-risk (pT1-2) patients were correctly identified as T1-2 on CT (Table 2), meaning that 18 (45%) cases were over-staged, and potentially over-treated with neoadjuvant therapy. The number of over-staged cases was reduced to 7 out of 35 (20%) in batches 3–4. In these last two batches, 53 out of 60 (88%) cases were correctly identified as high-risk colon cancer on CT, compared to 52 out of 70 (74%) cases in batches 0–2.

## N-staging

**Fig. 3:**
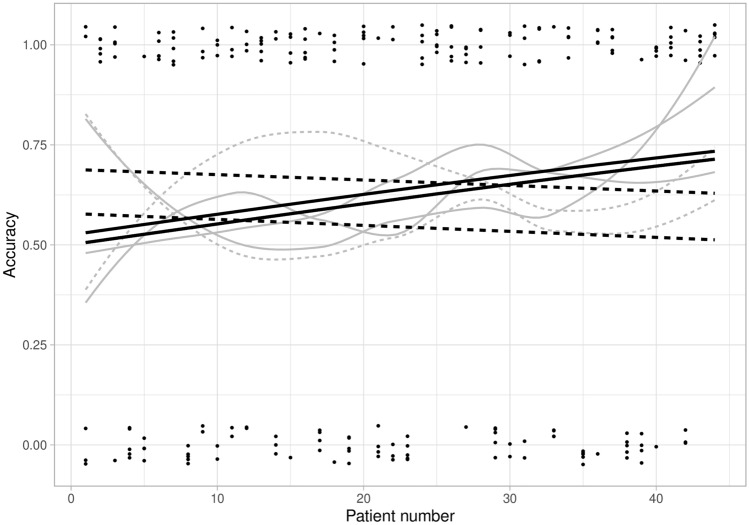
Learning curves for N-staging (N0 vs. N1-2) of colon cancer, separately for each radiologist. Accuracy of radiologists who received feedback (dashed line) and did not receive feedback (solid line) is presented. Accuracy was plotted and fitted using generalized estimating equations logistic regression models (black lines, the lower black line represents the same learning curve for two radiologists). Dots represent the number of correct (around 1.00) or incorrect (around 0.00) staged readings, and gray lines are non-parametric smoothed curves

**Fig. 4:**
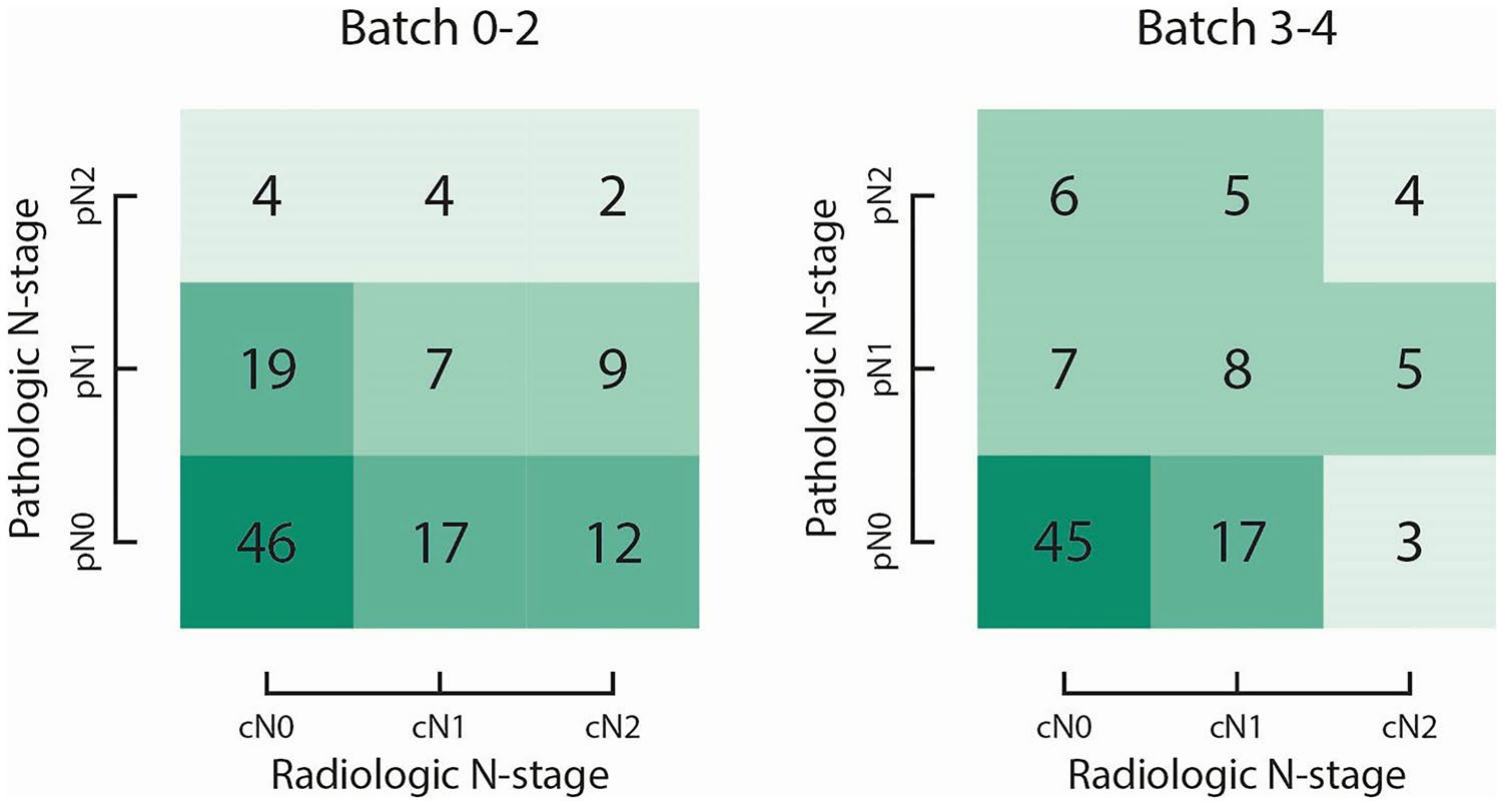
Contingency tables with radiologic assessment and pathologic assessment of lymph node involvement depicted for batch 0–2 and batch 3–4. The depicted numbers represent the number of readings

Table 2 summarizes the diagnostic performance of all radiologists in distinguishing N0 vs. N1-2. Modest improvement was seen in accuracy between batch 0–2 (57%, 68/120) and batch 3–4 (67%, 67/100) (*p* = 0.371). The learning curve for accuracy of assessing lymph node involvement in colon cancer is depicted in Fig. 3. There was no significant learning effect in either group. There was a minimal improvement in the overall concordance between radiologic N-stage and pathologic N-stage when comparing batch 0–2 (*κ* = 0.01) vs. batch 3–4 (*κ* = 0.27) (Fig. 4). Sensitivity improved significantly between batch 0 and batches 1–4 (*p* < 0.001), but only reached 60% in the final batch. Specificity increased slightly between batch 0–2 and batch 3–4 and reached 69% (*p* = 0.575).

### Pathologic characteristics of challenging cases

Pathologic characteristics of challenging cases were thoroughly reviewed. For T-staging, cases were selected in case of incorrect staging by 3 or more readers and/or the expert. Cases scored incorrectly by 4 or more readers and/or the expert were selected for N-staging. This resulted in 13 selected cases for T-stage and 17 for N-stage.

**Fig. 5:**
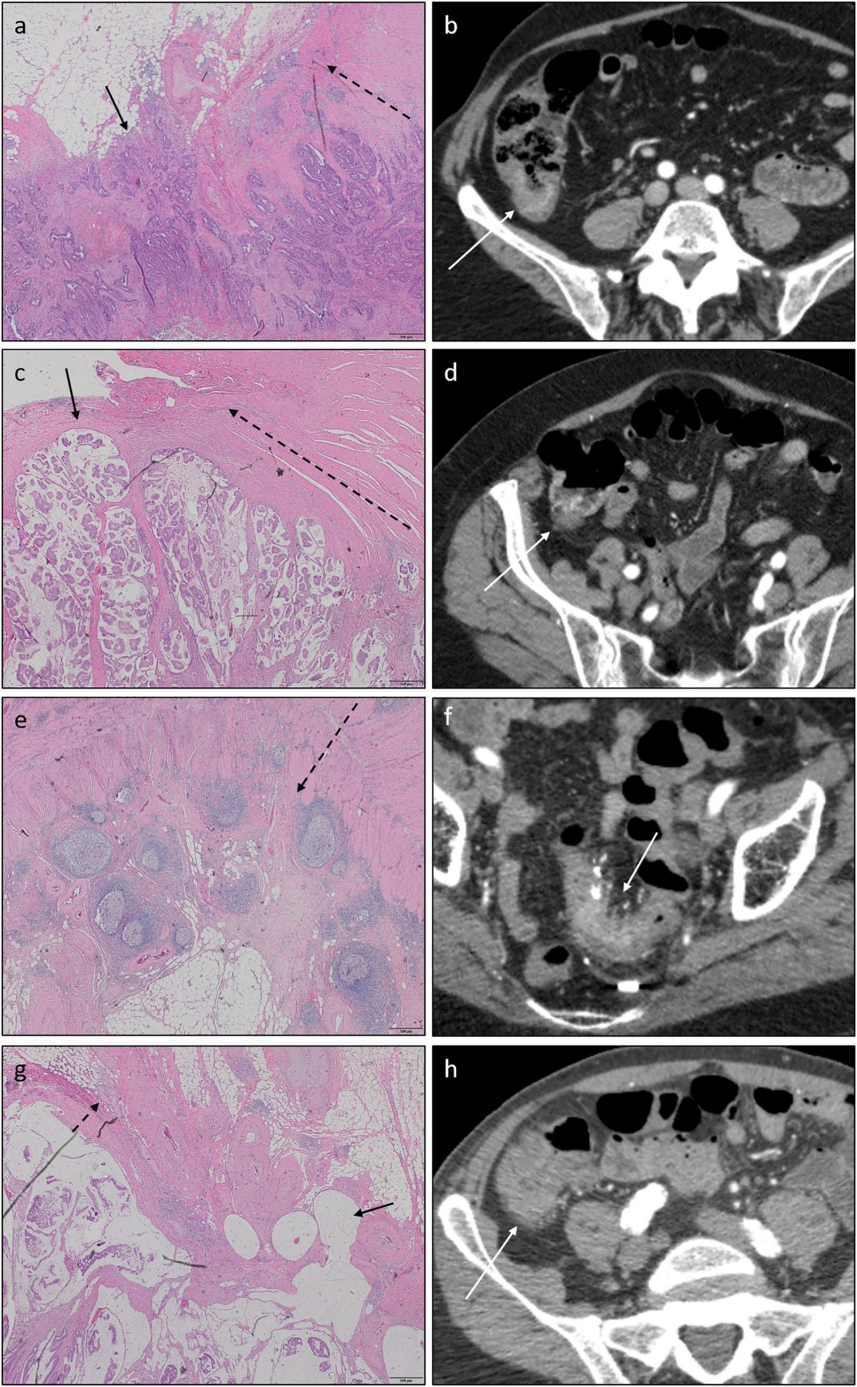
Four examples of challenging cases. Panels a + b, c + d, e + f, and g + h represent histology (Hematoxylin & Eosin stain, 2 × magnification) and radiology (axial CT image at tumor level) of corresponding cases. Dashed arrows indicate the muscularis propria and point toward the subserosa. Black arrows indicate invasive tumor area. White arrows point out the tumor on CT. **a** pT3 tumor with minimal invasion through the muscularis propria. No stromal or inflammatory reaction present in the surrounding subserosa. **b** Case was under-staged by 3 radiologists and the expert. **c** pT3 tumor with mucinous phenotype. Invasive part almost completely consists of mucus. **d** Case was under-staged by 4 radiologists and the expert. **e** pT2 tumor with a striking immune reaction in the subserosa at the tumor location. **f** Case was over-staged by 4 radiologists and the expert. **G** Example of a case with discordant assessment of pT-stage between initial evaluation and second review. Case was restaged from pT2 to pT3. **h** Case was scored as cT3 by three radiologists and as cT2 by 2 radiologists and the expert.

In two radiologically under-staged cases for primary tumor, the part invading through the muscularis propria was limited to less than 1 mm, without any stromal reaction in the surrounding subserosa (Fig. 5a + b). In 3 other under-staged cases, the invading part almost completely consisted of mucus, making it difficult to detect on CT (Fig. 5c + d). Review of the H&E sections in one over-staged case for primary tumor demonstrated a significant inflammatory reaction in the adjacent subserosa, which probably led to false-positive readings (Fig. 5e + f). Importantly, in 3 cases, the review by an expert pathologist led to a different T-stage from the initial pathology report, showing inter-observer discrepancy between pathologists. All three were restaged from pT2 to pT3 (Fig. 5g + h). The initial pT-stages were used as reference standard in all analysis. In the other 4 challenging cases, no pathologic explanation was found for incorrect staging on CT.

In 8 out of 17 challenging cases for N-staging, the pathology reports mentioned a possible explanation for incorrect radiologic staging. In 6 cases, the macroscopy section clearly indicated the presence of enlarged, suspicious lymph nodes, correlating with the enlarged, suspicious nodes identified on imaging. However, these were negative for metastases on microscopic examination. In two other cases, no suspicious lymph nodes were detected on CT and macroscopic evaluation also revealed no enlarged or suspicious lymph nodes. However, during microscopic examination, small metastases were found in 1 out of 12 examined lymph nodes in one case and in 3 out of 17 examined lymph nodes in the other case.

#### Confidence and feedback

Reader’s confidence was scored on a 0 to 4 scale with 4 as the most confident and 0 as the least confident. The average score for distinguishing T1-2 vs. T3-4 was 3.03 (standard error 0.66), while it was 3.08 (standard error 0.73) for N-staging. These scores correspond to being *probably certain* about staging. Reader’s confidence for distinguishing T1-2 vs. T3-4 colon cancer and lymph node involvement did not change significantly with increasing numbers of reviewed cases (Table 3). Importantly, we observed no significant difference in reader’s confidence between accurately and erroneously staged cases when combining all readers and batches (results not shown).

**Table 3:** Reader’s confidence in distinguishing T1-2 vs. T3-4 colon cancer and lymph node involvement using a 0–4 scale with 4 as most certain

Batch	All readers	With Feedback	Without feedback	*P* value
*T-staging (T1-2 vs. T3-4)*				
0	2.80 ± 0.50	2.70 ± 0.48	2.87 ± 0.52	0.239
1	3.02 ± 0.69	2.95 ± 0.76	3.07 ± 0.64	0.512
2	3.13 ± 0.73	2.56 ± 0.51	3.52 ± 0.58	< 0.001
3	3.14 ± 0.67	2.90 ± 0.72	3.30 ± 0.60	0.008
4	2.92 ± 0.63	2.60 ± 0.50	3.13 ± 0.63	0.003
All	3.03 ± 0.66	2.76 ± 0.62	3.21 ± 0.62	< 0.001
*N-staging (N0 vs. N1-2)*				
0	3.20 ± 0.82	3.00 ± 0.82	3.33 ± 0.82	0.174
1	2.96 ± 0.67	2.70 ± 0.73	3.13 ± 0.57	0.027
2	3.31 ± 0.70	2.78 ± 0.65	3.67 ± 0.48	< 0.001
3	3.00 ± 0.76	2.45 ± 0.51	3.37 ± 0.67	< 0.001
4	3.04 ± 0.73	2.65 ± 0.67	3.30 ± 0.65	< 0.001
All	3.08 ± 0.73	2.68 ± 0.67	3.36 ± 0.64	< 0.001

*Note* numbers are mean ± standard deviation. P values are derived from the comparison of feedback groups

The feedback group (*n* = 2) received information on the pathologic T- and N-stages after interpretation of each batch. This provided them with the opportunity to compare the pathologic stages with their radiologic findings. However, this did not result in a statistically significant difference in diagnostic performance for T- and N-staging compared to the group with no feedback (Figs. 1 and 3). Nonetheless, feedback did influence the reported confidence scores for T- and N-staging, with a significantly lower overall confidence in the feedback group compared to the group without feedback, both for batches 2–4 and overall (Table 3).

#### Reading time

The median reading time of all readers for batch 1–4 was 4 min (range 2—15 min). Reading time decreased significantly with increasing number of reviewed cases for all readers between batch 1 and 2–4 (*p* = 0.017), between batch 1–2 and 3–4 (*p* = 0.001), and between batch 1–3 and 4 (*p* < 0.001). The group receiving feedback had a significantly longer reading time than the group without feedback (*p* < 0.001) (Table 4).

**Table 4:** Comparison of reading time in minutes for the different batches

Batch	All readers	With Feedback	Without feedback	*P* value
1	5.5 (2.0–15.0)	7.0 (2.8–15.0)	5.3 (2.0–13.0)	< 0.001
2	4.7 (2.0–14.5)	6.1 (2.5–14.5)	4.0 (2.0–9.7)	< 0.001
3	4.0 (2.0–12.7)	5.6 (2.0–12.7)	3.9 (2.4–6.7)	< 0.001
4	3.6 (2.0–9.6)	3.9 (2.0–9.6)	3.5 (2.0–6.0)	< 0.001
All	4.0 (2.0–15.0)	5.4 (2.0–15.0)	4.0 (2.0–13.0)	< 0.001

*Note* numbers are median (range). P values are derived from the comparison of feedback groups

## Discussion

This study evaluated the accuracy and learning curve for locoregional staging of colon cancer patients on CT by experienced radiologists into the context of selecting high-risk patients for neoadjuvant therapy. The diagnostic performance in distinguishing T1-2 vs. T3-4 improved significantly with increasing number of reviewed cases. We further identified pathologic traits as potential explanations for challenging cases, such as minimal invasion through the muscularis propria and invasion of a mucinous tumor component for radiologically under-staged cases, and extensive immune reaction in the subserosa adjacent to the tumor for radiologically over-staged cases.

The accuracy in distinguishing T3-4 colon cancer improved from 60 to 80% as the number of reviewed cases increased. The accuracy for the final 10 cases observed in this study is comparable with the previous published meta-analysis of Nerad et al., which reported an overall accuracy of 82% in distinguishing T1-2 vs. T3-4 [4]. Interestingly, specificity in distinguishing T1-2 vs. T3-4 improved drastically after training and reached 85% in batch 4, compared to that of 70% from the same meta-analysis [4]. Besides the limited sample size, an explanation could be the definition of radiologic high T-stage tumors used in this study. We used only smooth or nodular, and not spiculated extension beyond the normal delineation of the bowel wall on CT as tumor extension beyond muscularis propria layer. This definition is now widely used to prevent false-positive cases caused by minimal pericolonic fat stranding due to benign desmoplastic reaction [12].

One important concern of neoadjuvant therapy in colon cancer patients is the adequacy of CT in selecting high-risk patients [2, 13]. False-positive readings on CT can result in subsequent exposure to unnecessary treatment and should therefore be minimized. It is, therefore, important to note that in this study, incorrect staging was more frequently caused by under-staging rather than over-staging. Under-staging is less detrimental than over-staging since under-staged cases will eventually be recognized as high-risk colon cancer during pathologic examination of the surgical specimen. In addition, all pT1 and pT4 cases were accurately identified on CT as low-risk and high-risk cases, respectively. Inaccurate staging was solely caused by the distinction between pT2 and pT3 disease. Moreover, the high specificity for T-staging reached in our study shows that training and repetition can ensure a high validity for patient selection for neoadjuvant therapy on CT by experienced radiologists. As opposed to T-staging, specificity for distinguishing N0 vs. N1-2 only improved to a level of 63%, meaning that patient selection for neoadjuvant chemotherapy based on N-staging harbors a great risk of mis-staging. This risk could not be minimized with training and practice.

All scans were additionally scored by an expert radiologist. The expert performance for T-staging was consistent over all batches with an overall accuracy of 80%. This indicates that the observed improvement in diagnostic performance of all readers was not influenced by differences in case difficulty between batches. In addition, our approach enabled all readers to reach a comparable diagnostic accuracy for locoregional staging in colon cancer that matches the expert level. Although the accuracy of differentiating T3-4 colon cancer on CT increased significantly after practice, it did not exceed 82%, still leaving a fair fraction of inaccurately staged cases. We reviewed the pathology reports and H&E-stained sections of challenging cases (incorrect staging by ≥ 3 radiologists and/or the expert) to identify potential pitfalls. This resulted in a possible explanation for incorrect radiologic staging in 9 out of 13 selected cases. Two explanations were found for false-negative readings: 1) a very limited part invading through the muscularis propria, explaining 2/7 under-staged cases; and 2) an invading part that almost completely consists of mucus, explaining 3/7 under-staged cases. Resolution of the CT appeared to be insufficient to recognize these features in the absence of a stromal or inflammatory reaction in the surrounding subserosa. On the other hand, one false-positive case could be explained by a striking inflammatory reaction in the subserosa without tumor invasion through the muscularis propria. The differentiation between an inflammatory response and tumor invasion is a well-known problem in CT staging of colon cancer [14]. Importantly, re-evaluation of H&E-stained sections resulted in a discordant assessment of the tumor invasion depth with the initial evaluation in three cases. The initial T-stages were used as reference standard in all analysis. These findings demonstrate inter-reader discrepancy between pathologists, showing that not only radiological, but also microscopic interpretation of colon cancer T-staging can be challenging.

No improvement was seen in the detection of lymph node involvement on CT, which is in line with our previous observations [8]. N-staging remains unreliable in selecting high-risk patients for neoadjuvant therapy [4, 7, 15–17], and the results of this study suggest that there is no or minimal improvement of experienced radiologists’ performance to adequately determine lymph node status of colon cancer even after training and practice. Interestingly, review of the pathology reports of challenging cases (incorrect staging by ≥ 4 radiologist and/or the expert) showed a correlation between radiologic assessment and macroscopic examination. Both radiology and macroscopic evaluation use size as a main determinant of suspected lymph node involvement. Our results demonstrate that size-dependent evaluation of lymph nodes leads to false-negative readings in the presence of microscopic metastases and false-positive readings in inflammation-induced enlargement. This is further supported by a recent paper, showing that radiologic assessment of lymph node status is even more complicated in patients with deficient mismatch repair (dMMR) colon cancer [18]. dMMR tumors are known to induce greater inflammatory responses, resulting in immune cell infiltration and enlargement of lymph nodes [19].

Interestingly, significant longer reading times and lower confidence in T- and N-staging were demonstrated in the group receiving feedback. Feedback seems to have raised awareness of imaging limitations and led readers into a more detailed assessment to gather subtle clues on locoregional staging. Importantly, feedback did not improve reader’s ability to recognize difficult cases. In both the feedback and no-feedback group, no difference was observed in reader’s confidence between accurately and erroneously staged cases.

The diagnostic performance of CT in selecting high-risk colon cancer patients for neoadjuvant therapy is often solely based on its ability to predict pathologic stage. As shown by the pathology review of challenging cases, some features determining pathologic T-stages of colon cancer are difficult to be adequately recognized on CT. Radiologic T- and N-stages in patients with non-metastatic colon cancer have been shown to be independent prognostic factors for overall and disease-free survival [20]. The 5-year overall survival rates of patients with radiologic TNM stages I, II, and III were 90%, 81%, and 70%, respectively. Application of alternative and advanced radiologic features, such as radiomics or deep-learning techniques, could potentially improve the identification of high-risk features of colon cancers on CT for better patient stratification. Future research in large cohorts with sufficient follow-up data is warranted.

There are several limitations to this study. First, this was a retrospective study that utilized scans from a single center. Second, the number of reviewed cases utilized for analysis limits the ability for subgroup analysis using various parameters. Third, we only utilized 3-mm thickness scans, and imaging acquisition parameters were heterogeneous in the study cohort. We believe that the heterogeneous imaging acquisition parameters can have a value in generalizability of our results, but future studies with various slice thickness CT scans should be done to evaluate the effect of CT slice thicknesses. Last, we selected scans with optimal image quality which might result in an over-estimation of the diagnostic performance. Nevertheless, we believe that this was important to match current radiological standards.

In conclusion, this study shows that experienced radiologists were able to reach an adequate diagnostic performance in locoregional staging of colon cancer patients on CT after training and repetition of radiological staging. Importantly, the T-staging specificity level reached indicates a high validity in patient selection for neoadjuvant therapy on CT. We also noted and identified pathologic features that can explain radiologic-pathologic discrepancies. Future studies can be done focusing on identifying additional radiologic features that might predict patient outcome and response to neoadjuvant therapy.
